# Chlorido[1,1′-(5-methyl-1,3-phenyl­ene)bis­(3,5-dimethyl-1*H*-imidazol-2-yl­idene)]platinum(II)

**DOI:** 10.1107/S1600536813006545

**Published:** 2013-03-16

**Authors:** Kui-Juan Peng, Zi-Xing Wang, Wen Wan

**Affiliations:** a Department of Chemistry, Shanghai University, Shanghai 200444, People’s Republic of China; b Key Laboratory of Advanced Display and System Applications, Ministry of Education, Shanghai University, Shanghai 200072, People’s Republic of China

## Abstract

In the title compound, [Pt(C_17_H_19_N_4_)Cl], the Pt^II^ cation is *C*,*C*′,*C*′′-chelated by the 1,1′-(5-methyl-1,3-phenyl­ene)bis­(3,5-dimethyl-1*H*-imidazolyl­idene) anion and coordinated by a Cl^−^ anion in a distorted square-planar coordination geometry. π–π stacking is observed between nearly parallel imidazole and benzene rings of adjacent mol­ecules, the centroid–centroid distance being 3.802 (4) Å.

## Related literature

For the application of Pt^II^ complexes in organic light-emitting diodes, see: Yang *et al.* (2008 ▶); Bakken *et al.* (2012 ▶); Fleetham *et al.* (2012 ▶). For a related compound, see: Wang *et al.* (2010 ▶).
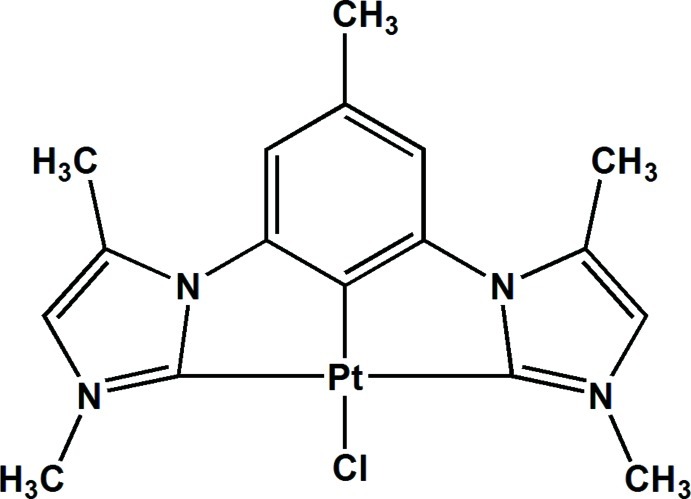


## Experimental

### 

#### Crystal data

[Pt(C_17_H_19_N_4_)Cl]
*M* *_r_* = 509.90
Monoclinic, 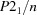

*a* = 11.042 (5) Å
*b* = 14.552 (6) Å
*c* = 11.524 (5) Å
β = 116.049 (4)°
*V* = 1663.6 (12) Å^3^
*Z* = 4
Mo *K*α radiation
μ = 8.60 mm^−1^
*T* = 296 K
0.16 × 0.13 × 0.07 mm

#### Data collection

Bruker APEXII CCD diffractometer
Absorption correction: multi-scan (*SADABS*; Bruker, 2001 ▶) *T*_min_ = 0.340, *T*_max_ = 0.584
8411 measured reflections
2958 independent reflections
2579 reflections with *I* > 2σ(*I*)
*R*_int_ = 0.026

#### Refinement

*R*[*F* ^2^ > 2σ(*F* ^2^)] = 0.029
*wR*(*F* ^2^) = 0.080
*S* = 1.07
2958 reflections
189 parameters
H-atom parameters constrained
Δρ_max_ = 1.78 e Å^−3^
Δρ_min_ = −2.04 e Å^−3^

### 

Data collection: *APEX2* (Bruker, 2007 ▶); cell refinement: *SAINT* (Bruker, 2007 ▶); data reduction: *SAINT*; program(s) used to solve structure: *SHELXTL* (Sheldrick, 2008 ▶); program(s) used to refine structure: *SHELXTL*; molecular graphics: *SHELXTL*; software used to prepare material for publication: *SHELXTL*.

## Supplementary Material

Crystal structure: contains datablock(s) I, global. DOI: 10.1107/S1600536813006545/xu5680sup1.cif

Structure factors: contains datablock(s) I. DOI: 10.1107/S1600536813006545/xu5680Isup2.hkl

Additional supplementary materials:  crystallographic information; 3D view; checkCIF report

## Figures and Tables

**Table 1:** Selected bond lengths (Å)

Pt1—C1	1.909 (6)
Pt1—C9	2.045 (6)
Pt1—C10	2.040 (6)
Pt1—Cl1	2.4295 (19)

**Fig. 1.**
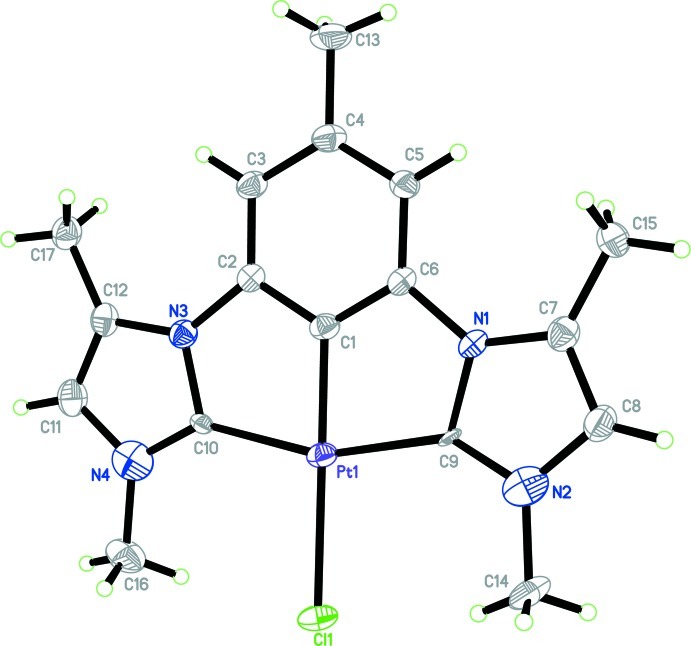
The molecular structure of the title compound, showing 50% probability displacement ellipsoids (arbitrary spheres for H atoms)

## supplementary crystallographic information

### Comment

Square planar Pt(II) complexes have attracted much attention due to their potential application in organic light-emitting diodes (Yang *et al.*, 2008). Pt(N—C—N)*X* (where *N*—*C*—*N*,di(2-pyridinyl)benzene-based tridentate ligands, *X*=monoanionic ligands), are reported to have much higher quantum yield than Pt(C—N)(LX) (where *C*—*N*,2-pyridylphenylbased bidentate ligands, LX, monoanionic ancillary ligands) with similar structures (Wang *et al.*, 2010), so designing and studying tridentate Pt complexes for application in blue and white phosphorescent OLEDs is a worthwhile undertaking (Fleetham *et al.*, 2012). Replacing pyridinyl group with methyl-imidazolyl group could potentially weaken the intermolecular interaction, resultingin a blue-shifted excimer emission (Bakken *et al.*,2012). Here we report a new Pt(C—C—C)*X* type of Pt complex using methyl-imidazolyl as ligands, structure shown in Figure 1.

In the title molecule the Pt(1)-C(1)-C(9) plane is almost coplanar with the benzene ring and with the plane of C(9)-N(1)-N(2) ring, the dihedral angles being 1.57° and 2.00°, respectively. The other Pt1 ring (Pt(1)-C(1)-C(10)) is, however, is slightly different, makes dihedral angles of 1.28° and 2.00° with the benzene ring and the plane of C(10)-N(3)-N(4), respectively. The Pt(II) centre forms a distorted square planar and makes an angle of 1.95° between the Pt(1)-C(1)-C(10) ring and Pt(1)-C(1)-C(9) ring; the bond lengths of Pt(1)—C(9) and Pt(1)—C(10) are almost the same, being 2.040 (4) and 2.045 (4), respectively. The angles of C(1)-Pt(1)-C(9) and C(1)-Pt(1)-C(10) being 79.38 (19)° and 79.62 (19)°, are also very near.

### Experimental

A mixture of 1,1'-[5-methyl-1,3-phenylene]bis[3,5-dimethyl-1*H*-imidazolium] diiodide (1 mmol) and 0.5 mmol silver oxide was stirred in a solution of 100 mL acetonitrile for 5 h at room temperature before 1equiv. Platinum chloride and 1eq. potassium carbonate were added. The reaction mixture was heated to reflux for an additional 24 h. Then the mixture was cooled to room temperature before 100 mL water was added. The resulting yellow precipitate was filtered off and washed with excessive methanol, water, and ether and dried under vacuum. The light yellowish product (in 20% yield) was obtained after thermal evaporation under high vacuum.^1^H NMR (500 MHz, δ in p.p.m., DMSO): 6.84 (s, 2H), 6.02 (s, 2H), 2.77 (s, 6H), 2.71(s, 6H), 2.35 (s, 3H).

### Refinement

Methyl H atoms were placed in calculated positions with C—H = 0.96 Å and torsion refined to fit the electron density with *U*_iso_(H) = 1.5*U*_eq_(C). Other H atoms were placed in calculated positions with C—H = 0.93 Å and refined in riding mode, *U*_iso_(H) = 1.2*U*_eq_(C).

### Crystal data

**Table:**

[Pt(C_17_H_19_N_4_)Cl]	*F*(000) = 976
*M**_r_* = 509.90	*D*_x_ = 2.036 Mg m^−^^3^
Monoclinic, *P*2_1_/*n*	Mo *K*α radiation, λ = 0.71073 Å
Hall symbol: -P 2yn	Cell parameters from 4377 reflections
*a* = 11.042 (5) Å	θ = 2.4–27.5°
*b* = 14.552 (6) Å	µ = 8.60 mm^−^^1^
*c* = 11.524 (5) Å	*T* = 296 K
β = 116.049 (4)°	Block, colorless
*V* = 1663.6 (12) Å^3^	0.16 × 0.13 × 0.07 mm
*Z* = 4	

### Data collection

**Table:**

Bruker APEXII CCD diffractometer	2958 independent reflections
Radiation source: fine-focus sealed tube	2579 reflections with *I* > 2σ(*I*)
Graphite monochromator	*R*_int_ = 0.026
Detector resolution: 10 pixels mm^-1^	θ_max_ = 25.0°, θ_min_ = 2.1°
φ and ω scans	*h* = −12→13
Absorption correction: multi-scan (*SADABS*; Bruker, 2001)	*k* = −16→17
*T*_min_ = 0.340, *T*_max_ = 0.584	*l* = −12→13
8411 measured reflections	

### Refinement

**Table:**

Refinement on *F*^2^	Primary atom site location: structure-invariant direct methods
Least-squares matrix: full	Secondary atom site location: difference Fourier map
*R*[*F*^2^ > 2σ(*F*^2^)] = 0.029	Hydrogen site location: inferred from neighbouring sites
*wR*(*F*^2^) = 0.080	H-atom parameters constrained
*S* = 1.07	*w* = 1/[σ^2^(*F*_o_^2^) + (0.0412*P*)^2^ + 5.3708*P*] where *P* = (*F*_o_^2^ + 2*F*_c_^2^)/3
2958 reflections	(Δ/σ)_max_ = 0.001
189 parameters	Δρ_max_ = 1.78 e Å^−^^3^
0 restraints	Δρ_min_ = −2.04 e Å^−^^3^

### Special details

**Table:**

Geometry. All e.s.d.'s (except the e.s.d. in the dihedral angle between two l.s. planes) are estimated using the full covariance matrix. The cell e.s.d.'s are taken into account individually in the estimation of e.s.d.'s in distances, angles and torsion angles; correlations between e.s.d.'s in cell parameters are only used when they are defined by crystal symmetry. An approximate (isotropic) treatment of cell e.s.d.'s is used for estimating e.s.d.'s involving l.s. planes.
Refinement. Refinement of *F*^2^ against ALL reflections. The weighted *R*-factor *wR* and goodness of fit *S* are based on *F*^2^, conventional *R*-factors *R* are based on *F*, with *F* set to zero for negative *F*^2^. The threshold expression of *F*^2^ > σ(*F*^2^) is used only for calculating *R*-factors(gt) *etc*. and is not relevant to the choice of reflections for refinement. *R*-factors based on *F*^2^ are statistically about twice as large as those based on *F*, and *R*- factors based on ALL data will be even larger.

### Fractional atomic coordinates and isotropic or equivalent isotropic displacement parameters (Å^2^)

**Table:**

	*x*	*y*	*z*	*U*_iso_*/*U*_eq_	
Pt1	0.48107 (2)	0.137708 (16)	0.63220 (2)	0.02999 (11)	
C1	0.6140 (6)	0.1354 (4)	0.5684 (6)	0.0310 (14)	
C2	0.6974 (6)	0.0590 (4)	0.5917 (6)	0.0296 (13)	
C3	0.7964 (6)	0.0559 (4)	0.5471 (6)	0.0345 (14)	
H3	0.8522	0.0049	0.5631	0.041*	
C4	0.8098 (7)	0.1319 (4)	0.4772 (7)	0.0390 (16)	
C5	0.7255 (6)	0.2074 (4)	0.4513 (6)	0.0335 (14)	
H5	0.7340	0.2566	0.4039	0.040*	
C6	0.6260 (6)	0.2085 (4)	0.4978 (6)	0.0287 (13)	
C7	0.5042 (7)	0.3599 (4)	0.4217 (7)	0.0387 (16)	
C8	0.3955 (7)	0.3958 (5)	0.4341 (7)	0.0450 (17)	
H8	0.3540	0.4519	0.4023	0.054*	
C9	0.4420 (6)	0.2595 (4)	0.5339 (6)	0.0340 (7)	
C10	0.5694 (6)	0.0138 (4)	0.7018 (6)	0.0362 (7)	
C11	0.6528 (8)	−0.1257 (5)	0.7757 (7)	0.0462 (18)	
H11	0.6655	−0.1825	0.8164	0.055*	
C12	0.7198 (7)	−0.0930 (4)	0.7090 (6)	0.0362 (14)	
C13	0.9173 (9)	0.1298 (6)	0.4307 (10)	0.060 (2)	
H13A	0.9079	0.1825	0.3773	0.091*	
H13B	0.9082	0.0749	0.3815	0.091*	
H13C	1.0045	0.1308	0.5035	0.091*	
C14	0.2445 (8)	0.3377 (6)	0.5408 (9)	0.056 (2)	
H14A	0.1713	0.2998	0.4838	0.084*	
H14B	0.2146	0.4003	0.5341	0.084*	
H14C	0.2744	0.3167	0.6281	0.084*	
C15	0.5829 (8)	0.4025 (5)	0.3576 (8)	0.053 (2)	
H15A	0.6767	0.4044	0.4175	0.080*	
H15B	0.5510	0.4639	0.3308	0.080*	
H15C	0.5714	0.3667	0.2836	0.080*	
C16	0.4651 (9)	−0.0611 (6)	0.8318 (8)	0.057 (2)	
H16A	0.4607	−0.0018	0.8661	0.085*	
H16B	0.4962	−0.1056	0.9003	0.085*	
H16C	0.3771	−0.0783	0.7677	0.085*	
C17	0.8297 (8)	−0.1388 (4)	0.6863 (8)	0.0439 (17)	
H17A	0.8081	−0.1359	0.5961	0.066*	
H17B	0.8375	−0.2019	0.7130	0.066*	
H17C	0.9136	−0.1078	0.7353	0.066*	
Cl1	0.3130 (2)	0.14031 (13)	0.7148 (2)	0.0560 (5)	
N1	0.5316 (5)	0.2784 (4)	0.4818 (5)	0.0340 (7)	
N2	0.3591 (5)	0.3319 (4)	0.5039 (5)	0.0340 (7)	
N3	0.6696 (5)	−0.0091 (4)	0.6648 (5)	0.0362 (7)	
N4	0.5613 (5)	−0.0576 (4)	0.7712 (5)	0.0362 (7)	

### Atomic displacement parameters (Å^2^)

**Table:**

	*U*^11^	*U*^22^	*U*^33^	*U*^12^	*U*^13^	*U*^23^
Pt1	0.02833 (16)	0.03514 (16)	0.03016 (16)	−0.00059 (10)	0.01622 (11)	−0.00590 (10)
C1	0.025 (3)	0.035 (3)	0.033 (3)	0.000 (3)	0.013 (3)	−0.007 (3)
C2	0.032 (3)	0.027 (3)	0.029 (3)	0.003 (3)	0.013 (3)	0.000 (2)
C3	0.032 (3)	0.036 (3)	0.037 (4)	0.008 (3)	0.017 (3)	0.003 (3)
C4	0.034 (4)	0.048 (4)	0.041 (4)	0.007 (3)	0.021 (3)	0.002 (3)
C5	0.035 (3)	0.033 (3)	0.035 (3)	0.002 (3)	0.018 (3)	0.004 (3)
C6	0.031 (3)	0.026 (3)	0.027 (3)	0.005 (2)	0.012 (3)	−0.001 (2)
C7	0.041 (4)	0.039 (4)	0.035 (4)	0.004 (3)	0.016 (3)	0.002 (3)
C8	0.048 (4)	0.038 (4)	0.049 (4)	0.014 (3)	0.022 (4)	0.004 (3)
C9	0.0303 (17)	0.0384 (17)	0.0344 (18)	0.0063 (14)	0.0151 (14)	−0.0035 (14)
C10	0.0376 (18)	0.0386 (18)	0.0329 (18)	−0.0016 (15)	0.0159 (15)	−0.0006 (14)
C11	0.051 (4)	0.041 (4)	0.039 (4)	0.000 (3)	0.012 (4)	0.008 (3)
C12	0.040 (4)	0.032 (3)	0.030 (3)	0.002 (3)	0.009 (3)	0.001 (3)
C13	0.053 (5)	0.069 (6)	0.081 (6)	0.021 (4)	0.050 (5)	0.019 (4)
C14	0.052 (5)	0.061 (5)	0.064 (5)	0.024 (4)	0.033 (4)	0.002 (4)
C15	0.067 (5)	0.046 (4)	0.062 (5)	0.017 (4)	0.042 (4)	0.024 (4)
C16	0.071 (5)	0.067 (5)	0.044 (4)	−0.012 (4)	0.036 (4)	0.001 (4)
C17	0.045 (4)	0.038 (4)	0.048 (4)	0.003 (3)	0.019 (4)	0.003 (3)
Cl1	0.0584 (12)	0.0608 (12)	0.0749 (14)	0.0069 (9)	0.0531 (12)	−0.0027 (9)
N1	0.0303 (17)	0.0384 (17)	0.0344 (18)	0.0063 (14)	0.0151 (14)	−0.0035 (14)
N2	0.0303 (17)	0.0384 (17)	0.0344 (18)	0.0063 (14)	0.0151 (14)	−0.0035 (14)
N3	0.0376 (18)	0.0386 (18)	0.0329 (18)	−0.0016 (15)	0.0159 (15)	−0.0006 (14)
N4	0.0376 (18)	0.0386 (18)	0.0329 (18)	−0.0016 (15)	0.0159 (15)	−0.0006 (14)

### Geometric parameters (Å, º)

**Table:**

Pt1—C1	1.909 (6)	C10—N3	1.389 (8)
Pt1—C9	2.045 (6)	C11—C12	1.366 (10)
Pt1—C10	2.040 (6)	C11—N4	1.400 (9)
Pt1—Cl1	2.4295 (19)	C11—H11	0.9300
C1—C6	1.380 (8)	C12—N3	1.345 (8)
C1—C2	1.392 (8)	C12—C17	1.504 (10)
C2—C3	1.398 (8)	C13—H13A	0.9600
C2—N3	1.419 (8)	C13—H13B	0.9600
C3—C4	1.414 (9)	C13—H13C	0.9600
C3—H3	0.9300	C14—N2	1.504 (9)
C4—C5	1.385 (9)	C14—H14A	0.9600
C4—C13	1.502 (10)	C14—H14B	0.9600
C5—C6	1.419 (8)	C14—H14C	0.9600
C5—H5	0.9300	C15—H15A	0.9600
C6—N1	1.409 (7)	C15—H15B	0.9600
C7—N1	1.339 (8)	C15—H15C	0.9600
C7—C8	1.372 (10)	C16—N4	1.507 (9)
C7—C15	1.499 (10)	C16—H16A	0.9600
C8—N2	1.399 (9)	C16—H16B	0.9600
C8—H8	0.9300	C16—H16C	0.9600
C9—N2	1.337 (8)	C17—H17A	0.9600
C9—N1	1.393 (8)	C17—H17B	0.9600
C10—N4	1.338 (8)	C17—H17C	0.9600
			
C1—Pt1—C10	79.6 (3)	C4—C13—H13A	109.5
C1—Pt1—C9	79.2 (2)	C4—C13—H13B	109.5
C10—Pt1—C9	158.8 (2)	H13A—C13—H13B	109.5
C1—Pt1—Cl1	179.6 (2)	C4—C13—H13C	109.5
C10—Pt1—Cl1	100.12 (18)	H13A—C13—H13C	109.5
C9—Pt1—Cl1	101.06 (17)	H13B—C13—H13C	109.5
C6—C1—C2	120.2 (6)	N2—C14—H14A	109.5
C6—C1—Pt1	120.1 (4)	N2—C14—H14B	109.5
C2—C1—Pt1	119.6 (5)	H14A—C14—H14B	109.5
C1—C2—C3	120.9 (6)	N2—C14—H14C	109.5
C1—C2—N3	112.1 (5)	H14A—C14—H14C	109.5
C3—C2—N3	127.1 (5)	H14B—C14—H14C	109.5
C2—C3—C4	118.5 (6)	C7—C15—H15A	109.5
C2—C3—H3	120.7	C7—C15—H15B	109.5
C4—C3—H3	120.7	H15A—C15—H15B	109.5
C5—C4—C3	121.0 (6)	C7—C15—H15C	109.5
C5—C4—C13	120.0 (6)	H15A—C15—H15C	109.5
C3—C4—C13	119.0 (6)	H15B—C15—H15C	109.5
C4—C5—C6	119.1 (6)	N4—C16—H16A	109.5
C4—C5—H5	120.4	N4—C16—H16B	109.5
C6—C5—H5	120.4	H16A—C16—H16B	109.5
C1—C6—N1	112.2 (5)	N4—C16—H16C	109.5
C1—C6—C5	120.2 (5)	H16A—C16—H16C	109.5
N1—C6—C5	127.6 (5)	H16B—C16—H16C	109.5
N1—C7—C8	107.0 (6)	C12—C17—H17A	109.5
N1—C7—C15	124.9 (6)	C12—C17—H17B	109.5
C8—C7—C15	128.1 (6)	H17A—C17—H17B	109.5
C7—C8—N2	107.0 (6)	C12—C17—H17C	109.5
C7—C8—H8	126.5	H17A—C17—H17C	109.5
N2—C8—H8	126.5	H17B—C17—H17C	109.5
N2—C9—N1	105.5 (5)	C7—N1—C9	110.9 (5)
N2—C9—Pt1	141.3 (5)	C7—N1—C6	133.9 (6)
N1—C9—Pt1	113.2 (4)	C9—N1—C6	115.1 (5)
N4—C10—N3	105.6 (5)	C9—N2—C8	109.6 (5)
N4—C10—Pt1	140.8 (5)	C9—N2—C14	122.4 (6)
N3—C10—Pt1	113.5 (4)	C8—N2—C14	128.0 (6)
C12—C11—N4	107.0 (6)	C12—N3—C10	110.7 (5)
C12—C11—H11	126.5	C12—N3—C2	134.3 (5)
N4—C11—H11	126.5	C10—N3—C2	115.1 (5)
N3—C12—C11	107.1 (6)	C10—N4—C11	109.6 (6)
N3—C12—C17	124.5 (6)	C10—N4—C16	123.4 (6)
C11—C12—C17	128.4 (6)	C11—N4—C16	127.0 (6)
